# Co-Administered Polymeric Nano-Antidotes for Improved Photo-Triggered Response in Glioblastoma

**DOI:** 10.3390/pharmaceutics10040226

**Published:** 2018-11-10

**Authors:** Janel Kydd, Rahul Jadia, Prakash Rai

**Affiliations:** 1 Biomedical Engineering and Biotechnology Program, University of Massachusetts Lowell, 1 University Ave, Lowell, MA 01854, USA; Janel_Kydd@student.uml.edu (J.K.); Rahul_Jadia@student.uml.edu (R.J.); 2 Department of Chemical Engineering, University of Massachusetts Lowell, 1 University Ave, Lowell, MA 01854, USA

**Keywords:** drug delivery, cancer, polymer, nanomedicine, angiogenesis, blood–brain barrier

## Abstract

Polymer-based nanoparticles (NPs) are useful vehicles in treating glioblastoma because of their favorable characteristics such as small size and ability to cross the blood–brain barrier, as well as reduced immunogenicity and side effects. The use of a photosensitizer drug such as Verteporfin (BPD), in combination with a pan-vascular endothelial growth factor receptor (VEGFR) tyrosine kinase inhibitor (TKI), Cediranib (CED), encapsulated in NPs will provide the medical field with new research on the possible ways to treat glioblastoma. Concomitant administration of BPD and CED NPs have the potential to induce dual photocytotoxic and cytostatic effects in U87 MG cells by (1) remotely triggering BPD through photodynamic therapy by irradiating laser at 690 nm and subsequent production of reactive oxygen species and (2) inhibiting cell proliferation by VEGFR interference and growth factor signaling mechanisms which may allow for longer progression free survival in patients and fewer systemic side effects. The specific aims of this research were to synthesize, characterize and assess cell viability and drug interactions for polyethylene-glycolated (PEGylated) polymeric based CED and BPD NPs which were less than 100 nm in size for enhanced permeation and retention effects. Synergistic effects were found using the co-administered therapies compared to the individual drugs. The major goal of this research was to investigate a new combination of photodynamic-chemotherapy drugs in nano-formulation for increased efficacy in glioblastoma treatment at reduced concentrations of therapeutics for enhanced drug delivery in vitro.

## 1. Introduction

Glioblastoma multiforme (GBM) accounts for approximately 40% of all high-grade, type IV, intracranial tumors [1]. GBM, a subtype of glioma, or glial cell origin tumor, affects the brain or spinal cord and is characterized by aggressive metastatic potential with poor survival outcomes of 14 months with traditional treatments [1]. Five in 100,000 persons are diagnosed per year with this fatal cancer [2]. Histological analysis of GBM tumors show finger-like astrocytes which extend into the deeper regions of the brain tissue, making surgical and adjuvant therapies futile [3]. Primary ablation of tumors is plagued by ultimate failure following 80–95% reoccurrence within 2 cm of the surgical resection margin and a mean time to progression of 8.6 months [4]. FDA-approved treatments, such as temozolomide, a DNA alkylating agent, or Bevacizumab, a monoclonal antibody which binds to vascular endothelial growth factor (VEGF), are typically given with concomitant radiation, though their efficacies have been mediocre at best [5,6,7]. Less invasive and tumor-targeted options are mandated, as unspecified cytotoxicity and deleterious side effects waiver a grim cost versus benefit in long term survival and quality of life for GBM patients [2,8,9].

Photodynamic therapy (PDT) is a minimally invasive procedure with a variety of applications, from dermatology to oncology, which uses light, oxygen and irradiation of a photosensitive drug at a certain wavelength [10,11]. Photosensitizers (PS) have also been shown to preferentially accumulate in tumor tissue cells, making them attractive candidates for cancer treatment [12,13,14,15]. Verteporfin (BPD) gets excited when irradiated with light at 690 nm, while simultaneously undergoing photo-induced reactions with molecular oxygen to create reactive oxygen species and induce localized cytotoxicity [16]. Photodynamic therapy has been proven efficacious in fluorescent image-guided surgical resection of brain tumors [4,17]. This method of treating glioma has extended the marginal edge of tumor removal by up to 10 mm, lending a 6 month improvement in overall survival gain [4]. A potential disadvantage of PDT is the vascular damage that may result which further enhances tumor reoccurrence and progression [18,19]. In addition, the criteria diagnosis of GBM is microvascular proliferation and tumor necrosis with abnormal vascular function and abundant angiogenesis [20,21,22]. Like all cytotoxic therapies, PDT stimulates the expression of hypoxia-inducible factor-1 alpha (HIF-1α) which increases secretion of vascular endothelial growth factor (VEGF), a key player in the formation of blood vessels and subsequent tumor regrowth [23]. A noteworthy fact to also take into consideration is the criteria diagnosis of GBM which includes microvascular proliferation and tumor necrosis with abnormal vascular function and abundant angiogenesis [20,21,22].

Following the photodestruction of tumor cells using PDT, the use of drugs that can block the upregulated VEGF interaction with its cognate receptor, VEGFR and the deleterious downstream signaling that can lead to cancer cell survival, metastasis and recurrence have improved treatment outcomes in several models of cancer [19,24,25,26,27]. Previous work showed decreased tumor volume and subsequent increased survival time when PDT and antiangiogenic therapy were given in combination, compared to the monotherapies alone [19,24,28].

**Figure 1:**
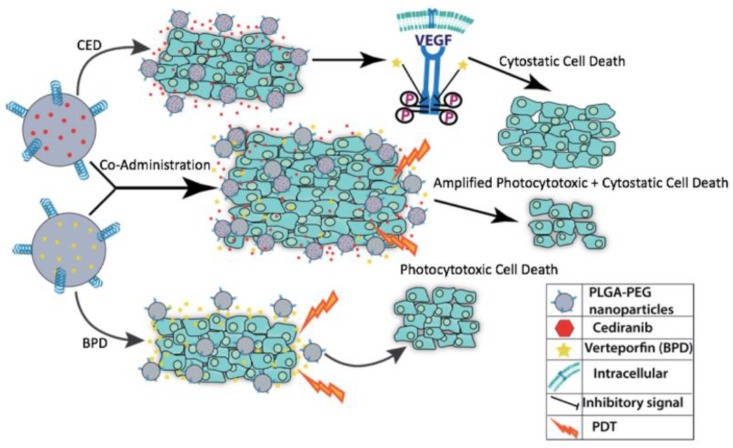
Cartoon schematic of the individual drug effects of Verteporfin (BPD) and Cediranib (CED) compared to the synergistic response of co-administration of both drugs to render increased cell death using these photocytotoxic and cytostatic compounds, respectively.

Combination therapy has been used across various cancer types in an effort to reduce the likelihood of multidrug resistance from efflux pumps and P-glycoproteins in the cell membrane of cancer cells, for example [29,30,31,32,33]. The tumor microenvironment is characterized by acidic pH, hypoxia and increased interstitial pressure, all of which promote the heterogeneous cell populations which are resistant, quickly adapting and mutating [34,35]. Dual tumor ablation can be achieved by using PDT-induced cytotoxicity combined with cytostatic drugs to improve the standard of care for GBM [10,36,37]. The study presented, shown in Figure 1, incorporated co-administration of BPD, an FDA-approved medication for age-related macular degeneration, known as Visudyne, and the pan-VEGFR tyrosine kinase inhibitor Cediranib (CED), an antiangiogenic compound with cytostatic properties [36,38,39,40,41,42,43,44,45]. A major advantage of using BPD as a PS is its longer wavelength activation which allows for better tumor penetration and reactive oxygen species generation while causing less peripheral tissue damage [45,46]. Furthermore, CED can target multiple VEGF receptors, lending its versatility in receptor tyrosine kinase inhibition [36]. The poor water solubility of each drug, however, is a major hindrance in their bioavailability. A drug delivery system using nanoencapsulation can protect the therapeutics from enzymatic degradation in the blood, while transporting a concentrated drug payload to the tumor tissue [47]. NPs which are surface modified with polyethylene glycol (PEG) additionally evade uptake by the reticular endothelial system (RES) and extend the circulation time of the drug in the body [48]. Tumors are noted for their leaky vasculature and enhanced permeation and retention (EPR), characteristics which allow for increased uptake of small molecules like NPs [49]. The blood–brain barrier (BBB) presents a difficult obstacle because of its tight fenestrations of capillaries [10]. NPs can, however, diffuse through the BBB, potentially reducing the toxic side effects associated with common therapeutics, and required effective dose necessitated for tumor cell death and unpleasant side effects of systemic free drug administration [50].

BPD and CED were tested in free and NP formulations as mono- and co-administered therapies in vitro using the U87 MG glioblastoma cell model. The particular combination chosen has not been studied thus far for glioblastoma treatment. The successful encapsulation of these hydrophobic compounds and synergistic effects found using concurrent delivery marks the novelty of our work which can be further tested to improve outcomes in GBM.

## 2. Materials and Methods

### 2.1. Materials

Acetone was ordered from Acros Organics, Waltham, MA, USA. Verteporfin was ordered from Sigma Aldrich, St. Louis, MO, USA. Cediranib was ordered from LC Labs, Woburn, MA, USA. Polymers, poly(lactide-co-glycolide)-b-poly(ethylene glycol) methyl ether (mPeg-PLGA) (MW 5:20 kD) and poly(lactic-co-glycolic acid) (PLGA) (MW 10:15 kD) were ordered from Polyscitech, West Lafayette, IN, USA. Verteporfin (BPD) was ordered from Sigma Adrich, St. Louis, MO, USA. All chemicals obtained were analytical grade and used without any further purification.

### 2.2. Methods

#### 2.2.1. Nanoparticle Synthesis

NPs were prepared using the solvent evaporation/precipitation technique [51,52]. The amount of drug used was 2% of the total polymer mass which consisted a blend of 75% PLGA and 25% mPEG-PLGA. The polymer concentration was 10 mg/mL. The polymers and drugs were dissolved in acetone completely prior to combining, followed by gentle vortexing. The organic phase (drugs and polymers) was slowly added dropwise to the aqueous phase (deionized water) in a 20-mL glass vial. The vial was left under stirring conditions (200 rpm) overnight for solvent evaporation. 24 h later, the sample was centrifuged at 800× *g* in a conical centrifugation tube for 10 min at 25 °C to remove unencapsulated drug.

#### 2.2.2. Characterization

**Size, PDI, and zeta potential:** The hydrodynamic diameter, polydispersity index (PDI), and zeta potential were measured using a dynamic light scattering (DLS) machine (Malvern Zetasizer Nano-ZS90, Malvern Instruments Limited, Worcestershire, UK). For DLS, 60 µL of the sample was diluted with 940 µL of deionized water in a plastic cuvette.

**Drug encapsulation efficiency:** The ThermoScientific NanoDrop 2000c machine (Thermo Fisher Scientific, Wilmington, DE, USA) was used to measure the absorbance of samples at 328 nm for CED and 688 nm for BPD by mixing 20 µL of the NP sample with 180 µL of 1% Triton-X in deionized water. Absorbance spectra along with compound structures can be found in Supplementary Figures S1 and S2. The encapsulation efficiency of the NPs was calculated using the drug loading efficiency Equation (1) below, whereby the initial synthesis weight of each drug loaded into NPs 0.2 mg, or 2% of the polymer weight (as previously mentioned in NP preparation).

(1) $$\mathrm{Encapsulation}\;\mathrm{Efficiency}\;\left(\%\right)=\frac{\mathrm{amount}\;\mathrm{of}\;\mathrm{drug}\;\mathrm{encapsulated}\;\left(\mathrm{mg}\right)}{\mathrm{initial}\;\mathrm{amount}\;\mathrm{of}\;\mathrm{drug}\;\mathrm{used}\;\mathrm{in}\;\mathrm{synthesis}\;\left(\mathrm{mg}\right)}\times 100\%,$$

**Scanning electron microscopy (SEM):** Polymeric NPs were diluted 100× in water and an aliquot was placed on a silicon wafer. To improve conductivity of the sample, gold sputtering was performed using with the Leica SCD500 Sputter Coater (Leica Microsystems, Bannockburn, IL, USA) [44]. Surface topography was assessed using a JEOL JSM 6390 Scanning Electron Microscope (JEOL USA, Peabody, MA, USA).

**Stability studies:** NPs were tested for stability based on size, polydispersity and zeta potential in a 1:1 ratio with EMEM media (supplemented with 10% fetal bovine serum and 1% penicillin/streptomycin) at 37 °C to simulate human body conditions. At selected time points, an aliquot was taken from each suspension and analyzed using DLS. The stability studies were performed over a 2-day period.

**Drug release kinetics:** Using the dialysis technique, 1 mL of NPs was transferred into 20 kDa MWCO dialysis membrane tubing (Spectrum Labs, Rancho Dominguez, CA, USA) and placed in a 1 L beaker of 1X PBS solution at 37 °C, pH 7.4 [53]. Aliquots of the NP solution were taken at set time points and checked for the concentration of CED and BPD using UV–Vis Spectroscopy at 320 nm and 688 nm, respectively. The release profile was repeated in triplicate for reproducibility.

#### 2.2.3. In Vitro Studies

**Monolayer Cell Culture:** Human glioblastoma cells, U87 MG, were obtained from and cultured using standard protocol provided by American Type Culture Collection (ATCC). The cells were cultured in EMEM media with 10% fetal bovine serum and 1% penicillin/streptomycin (Gibco, Life Technologies, Carlsbad, CA, USA). The cells were grown in an incubator under conditions of 5% CO_2_ and 37 °C.

**Imaging experiment:** The BPD fluorescence imaging experiment was conducted using monolayer cell culture of U87 MG cells. U87 MG cells were sub cultured in 35 mm single chambered dishes at a seeding density of 750,000 cells, followed by a 24-h incubation period. The following day, both cell types were dosed with free BPD and BPD NPs at concentrations of 25, 50, 100, 250 and 500 nM. Supplementary Figure S3 includes all concentrations tested, while the main text imaging results include 25 nM, 100 nM and 500 nM BPD for brevity. Following an incubation time of 60 min at 37 °C, 5% CO_2_, the dishes were washed twice with 1× PBS to remove excess BPD and imaged in media using the Cy5 filter in fluorescence microscopy (EVOS, FL, Electron Microscopy Sciences, Hatfield, PA, USA). This same procedure was used to image dishes which were concurrently dosed with CED and BPD free and NP formulations to assess whether CED inhibited BPD fluorescence. The concentrations used for each drug were 5 uM CED and 50 nM or 100 nM BPD. The average relative fluorescent intensity of each image was found using Image J software. The average pixel intensity of the control group (no treatment) was measured and subtracted from the average pixel intensity of each treatment arm. The results were presented as percent change (% ∆) in relative pixel (fluorescent) intensity compared to the control group (no treatment), given by Equation (2) below.

(2) $$\;\%\Delta =\frac{\mathrm{Fluorescent}\;\mathrm{Intensity}\;\mathrm{of}\;\mathrm{Treatment}\;\mathrm{Arm}-\mathrm{Fluorescent}\;\mathrm{Intensity}\;\mathrm{of}\;\mathrm{Control}\;\left(\mathrm{No}\;\mathrm{Treatment}\right)}{\mathrm{Fluorescent}\;\mathrm{Intensity}\;\mathrm{of}\;\mathrm{Control}\;\left(\mathrm{No}\;\mathrm{Treatment}\right)}\times 100\%$$

**Cell Viability Studies:** U87 MG cells were sub cultured in 35 mm dishes at a seeding density of 750,000 cells and incubated for 24 h at 37 °C, 5% CO_2_. Following 24-h incubation, cells were dosed with free BPD, free CED, free drug combination co-administration, and single and combination co-administered NPs at concentrations of 50 nM BPD and 2.5 uM and 5 uM CED in media for 1 h. Post 1 h incubation at 37 °C, 5% CO_2_, cells were rinsed twice in 1× PBS. Dark toxicity treatment arm dishes were rinsed twice with 1× PBS following drug incubation and media was replaced, followed by 24-h incubation at 37 °C, 5% CO_2_. Light treatment arm dishes were exposed to laser light at a wavelength of 690 nm, 2.5 J/cm^2^ light dose, power density of 100 mW/cm^2^ and 25 s irradiation time. Supplementary Figure S4 shows the set-up of the apparatus used for light irradiation of dishes. Post PDT, 1× PBS was replaced with media and cells were incubated for 24 h. Cell viability of dark and light toxicity treated dishes was assessed using the 1-(4,5-Dimethylthiazol-2-yl)-3,5-diphenylformazan (MTT) assay. Absorbance values were measured at 570 nm using the Molecular Devices SpectraMax M2 plate reader (Molecular Devices, LLC, Sunnyvale, CA, USA).

**Coefficient of Drug Interaction (CDI):** CDI was calculated using the formula given by CDI = AB/(A × B), where AB represents the ratio between the cell viability absorbance values of the co-administered drugs (CED + BPD) and control group, no treatment (dark toxicity), while A or B is the ratio between the cell viability absorbance values of each individual drug and the control group, no treatment (dark toxicity). The results were interpreted as CDI < 1 synergistic, CDI = 1 additive, CDI > 1 antagonistic [54].

**Statistical Analysis:** The results are presented as the average mean ± standard deviation for 3 independent repeats of experiments with triplicate samples in each group, unless stated otherwise. The statistical significance was evaluated using unpaired Student’s two-tailed *t*-test. *p* < 0.05 was considered statistically significant. *p* < 0.05 was represented as * and *p* < 0.01 as **.

## 3. Results

The size, polydispersity, zeta potential and drug encapsulation efficiency of BPD and CED single-loaded results are shown in Table 1 below. The overall sizes of both formulations were equal to or less than 100 nm, while the polydispersity index for BPD and CED NPs was approximately 0.1. The average zeta potentials were −24.1 mV and −16.3 mV, for BPD and CED, respectively. The encapsulation efficiency of BPD was slightly higher than CED, 87.7% versus 78.2%, respectively.

**Figure 2:**
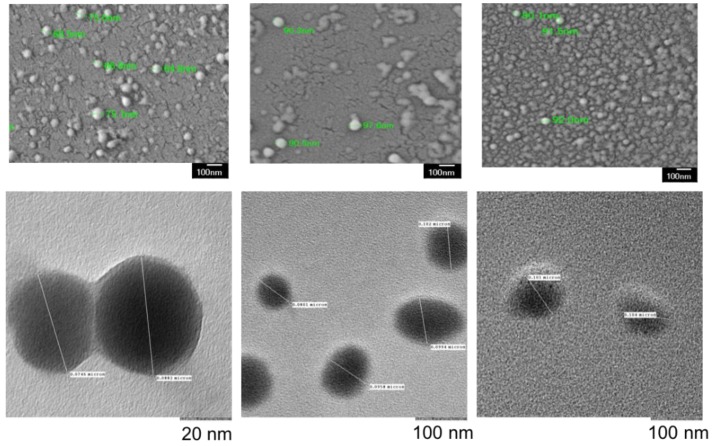
SEM (**top**) and TEM (**bottom**) images of EMPTY mPEG-PLGA (**left**), BPD (**middle**) and CED (**right**) NPs show uniform size distribution and spherical morphology. Empty NPs were slightly smaller than drug loaded NPs. Size measurements complemented DLS results. Small differences in size compared to DLS were attributed to dehydration of NPs during preparation for SEM and TEM. Scale bar: 100 nm.

Microscopy results, shown in Figure 2, yielded similar sizes in comparison to the DLS results, validating the findings of DLS and providing information about morphology (SEM).

**Figure 3:**
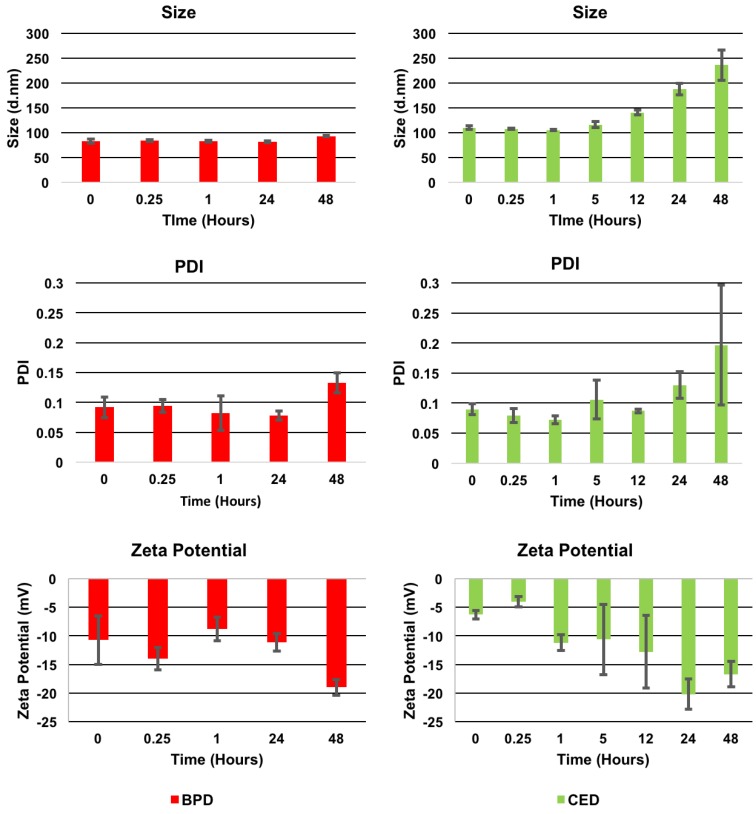
Stability study results (Size (**top**), PDI (**middle**), and Zeta Potential (**bottom**) for BPD (**left**) and CED (**right**) NPs over a 2-day period in media at 37 °C. Additional time points for CED NPs can be found at 5 and 12 h to show the gradual increase in size as NPs became unstable in media. Figure S5 in supplementary documentation also shows SEM imaging of CED NPs at 24 h to support the large size increase. Samples were tested in triplicate for reproducibility.

Stability studies, shown in Figure 3 below, demonstrated longer stability for BPD NPs which remained less than 100 nm. The PDI values were approximately 0.1 and zeta potential results ranged from −10 to −20 mV for the duration of the 48-h study in media. CED NPs were stable until 24 h while, thereafter, marked increases in size and PDI were observed. CED NPs gradually grew in size from 1 h on, where 5, 12 and 24 h showed an increase in size from 100 nm to about 200 nm. In addition, Supplementary Figure S5 shows SEM imaging of CED NPs at 24 h, concurring with the DLS results. At 48 h, the average size and PDI was more 250 nm and as high as 0.3, respectively, indicating instability and polydispersity for CED NPs.

**Figure 4:**
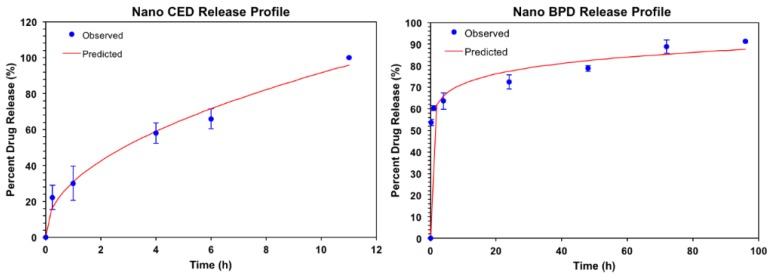
Release study results for CED NPs (**left**) over a 12-h period and BPD NPs (**right**) over a 4-day period in reverse dialysis 1× phosphate buffer solution at 37 °C, pH 7.4. Samples were tested in triplicate for reproducibility.

**Drug Release Kinetics:** The drug release kinetics were conducted at 37 °C in 1× PBS at pH 7.4, as shown in Figure 4 on the following page. Excel DD Solver was utilized to find the best fit for the release profiles using various drug delivery rate equations [55]. At 37 °C, the release profiles for BPD and CED at pH 7.4 best fit the Korsmeyer–Peppas (KP) model with an R^2^ value of 0.99 and 0.9846 for BPD and CED, respectively. The KP model describes drug dissolution from spherical polymeric drug delivery carriers using the power law mathematical model for one-dimensional release. Value “n” is the release exponent and “k_KP_” is the release constant, as indicated in the mathematical equation for % drug release using the KP model that follows.

(3) $$\;\%\;\mathrm{Drug}\;\mathrm{Release}\;\left(\mathrm{using}\;\mathrm{KP}\;\mathrm{power}\;\mathrm{law}\right)=\mathrm{kKP}\times t^{n},\;$$

Values of n ≤ 0.45 and 0.45 < n <0.89 indicate Fickian and non-Fickian diffusion, respectively [56]. The values for the constants and accompanying time to 50% and 80% drug release are shown in Table 2.

**Figure 5:**
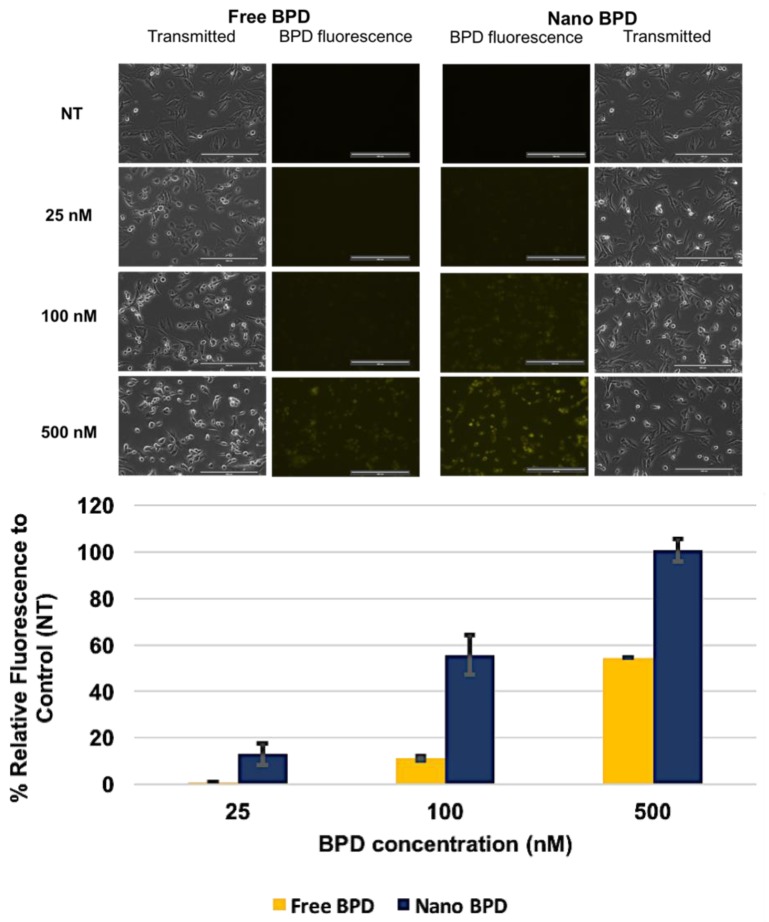
Imaging study results for free versus NP formulations of BPD using various concentrations of 25, 100, and 500 nM BPD to assess fluorescence in U87 cells. Images were taken at 20× magnification at 60% light intensity (Cy5 filter) for each image shown above. An increase in fluorescence is seen in the NP BPD form compared to the free drug, as shown in the bar graph for % fluorescent intensity relative to the control, no treatment (NT). Results are represented as mean ± standard deviation for *n* = 2. Scale Bar: 200 µm.

The release profiles of BPD and CED NPs are displayed in Figure 5. BPD had a more gradual release compared to CED, which had 100% drug release in under 12 h. BPD, however, had a burst release within the first 1.5 h, followed by steady, slower release of drug. The time to 50% drug release was noticeably different, whereby CED (Figure 4 (left side)) released at a much slower rate than BPD initially, more than 2 h to 50% release contrary to the 15 min for BPD, a rapid burst release effect as further visible in Figure 4 (right side). The time to 50% release is relevant to drug incubation period, as a one-hour time frame was used based on previous work with BPD. For the incubation period used in this study, it was ensured that sufficient BPD release occurred to elicit effective PDT cytotoxicity from accumulation of NPs within the cells by passive diffusion. CED had 30% drug release at the one-hour period. The gradual release for CED was sustained for an overall quick release time under 12 h for 100% drug release, while BPD presented gradual release over a 72-h period, indicating a biphasic profile. Relating the rates of release for each drug from the NPs, it appears that CED has a lower release rate constant, 58 approximately, than BPD, 31 approximately, however, CED has a much larger release exponent than BPD, 0.462 vs. 0.089, respectively. The time to 80% drug release was additionally much longer for BPD (36 h) compared to CED, 7.5 h. The drug release profiles may indicate that each drug has vastly different precipitation and degradation behavior. The release exponent show that BPD has Fickian diffusive properties, while CED has less-Fickian behavior.

**BPD Fluorescence Imaging Experiment:** Imaging was performed using fluorescent microscopy which showed cellular uptake of free drug versus NP BPD. The results for free vs NP 25, 100 and 500 nM BPD are displayed in Figure 5. Other concentrations of 50 and 250 nM BPD were also tested and are included in the supplementary information. BPD fluorescence was more pronounced at lower concentrations in the NP treatment groups, where 50 nM NP BPD showed fluorescence, while 250 nM free BPD (shown in Supplementary Figure S3) was the concentration at which free drug began to demonstrate marked fluorescence in cells. Image J analysis showed increases in fluorescence by 10%, 40% and 50% for NP compared to free drug at concentrations of 25, 100 and 500 nM BPD, found in the bar graph of Figure 5. The difference in fluorescence between free and NP was noteworthy.

Vibrant fluorescence for the BPD NP treatment groups increased dramatically as the concentration of BPD increased, while the free drug only showed noticeable differences in fluorescence at 250 and 500 nM (250 nM shown in Supplementary Figure S3). NP treatment also appeared to yield localized bright fluorescence within the cell organelles from the images obtained. These images were a critical portion of the study to understand the accumulation of NP in GBM cells in vitro.

**Figure 6:**
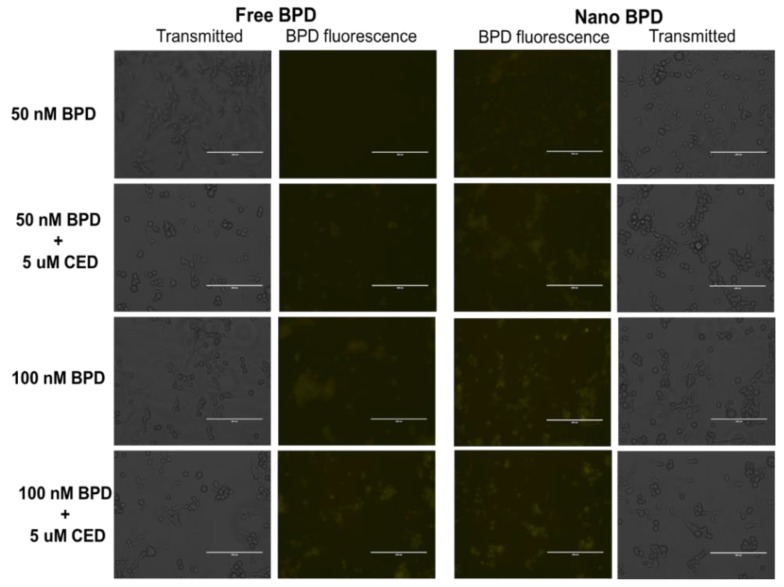
Imaging Study results for free (**left side**) versus NP (**right side**) formulations of BPD using various concentrations of 50 and 100 nM BPD to assess fluorescence in U87 cells. Cells were also treated with CED in certain treatment groups to assess the influence of CED on BPD fluorescence. Images were taken at 20× magnification at 60% light intensity (Cy5 filter) for each image shown above. An increase in fluorescence is seen in the NP BPD form compared to the free drug, while the incidence of CED does not appear to inhibit BPD fluorescence. Scale Bar: 200 µm.

CED Effect on the BPD Fluorescence Imaging Experiment: An additional imaging experiment was performed to assess the effect of CED on BPD fluorescence during co-administration and the results are shown in Figure 6 below. The concentrations tested for co-administration were 50 and 100 nM BPD, free drug and NP form, as well as co-administration with 5 uM CED, either in free or NP form. CED did not appear to dampen the fluorescence of BPD in either free drug or NP form. The images with and without co-administration of CED are similar in fluorescence.

**Figure 7:**
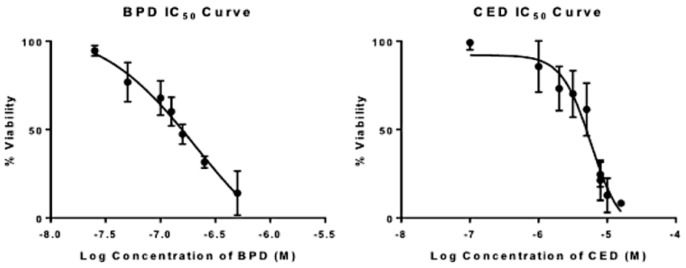
Effect of BPD and CED on U87 cellular survival as shown by IC_50_ results of free drugs BPD (left) and CED (right). GraphPad Prism log (inhibitor) vs response-variable slope (four parameters) non-linear regression analysis was used for *n* = 3 trials. The equation of the line is given as Y = Bottom + (Top-Bottom)/(1 + 10^((LogIC50 − X)*HillSlope)) based on GraphPad Prism analysis. Top and bottom refer to plateaus 100 and 0, respectively, on the y-scale for % Viability.

**Figure 8:**
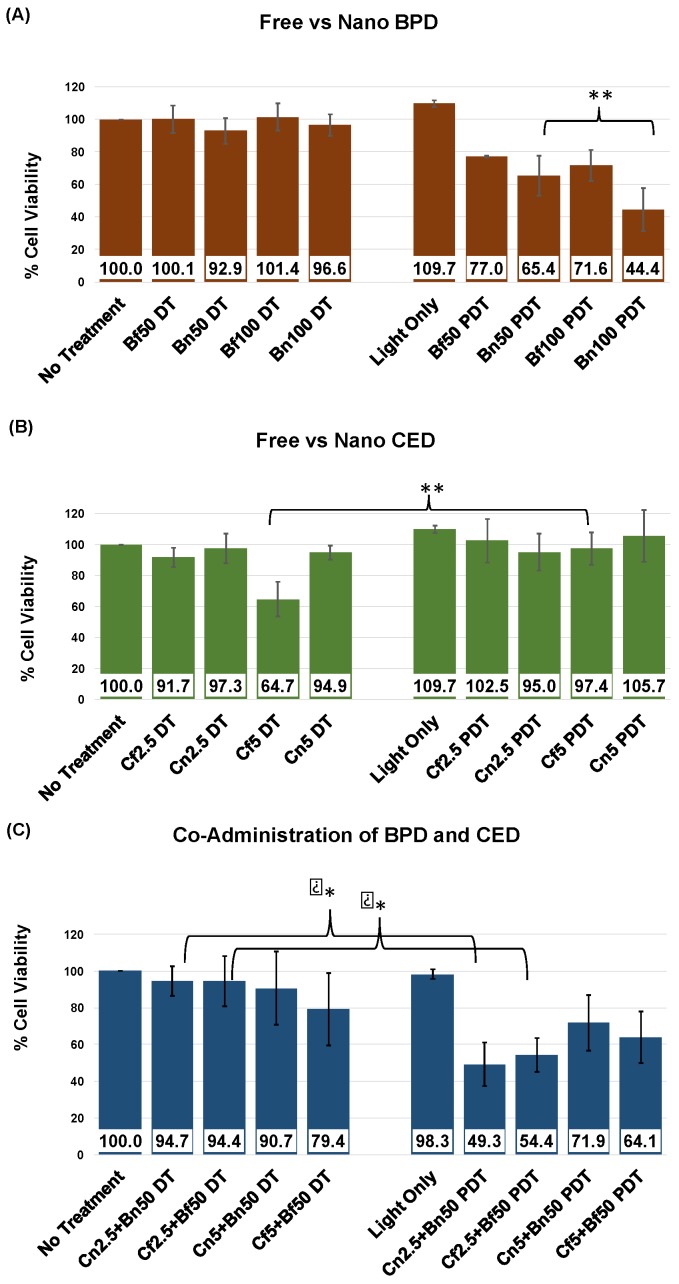
Effect of BPD and CED on U87 cellular survival as shown by free and NP formulation cell viability assay results of individual drugs, (**A**) BPD and (**B**) CED, at varied concentrations to assess optimal dose for cell death in the (**C**) co-administration of free or NP C2.5 or C5, and B50, simultaneous dosing where both CED and BPD were either in free or NP form, and incubation for 1 h. PDT treatment was consistent at 2.5 J/cm^2^, 100 mW/cm^2^, and 25 s exposure time. MTT assay analysis was performed at 24 h following PDT. All experiments shown were performed in triplicate. Differences with *p* < 0.05 were considered statistically significant using the Student’s *t*-test (* *p* <0.05, ** *p* < 0.01). DT, dark toxicity; PDT, photodynamic therapy; C, cediranib; B, bpd; f, free drug; n, NP formulation of drug; 2.5, 2.5 uM cediranib; 50, 50 nM bpd.

**Cell Viability Studies:** The killing efficacy of both free and NP BPD and CED were investigated in viability studies on U87 cells; results are shown in Figure 7 and Figure 8. The initial understanding of cell death caused by free drugs was found by a range of concentrations for each drug which were tested to assess the value at which 50% cell death occurs, or IC_50_ dose. The IC_50_ values for BPD and CED, respectively, were 172 nM and 6 uM, as shown in Figure 7 and Table 3 using non-linear regression analysis for a dose-response in GraphPad Prism software. The concentrations used to test free versus NP in single drug administration were gathered from the IC_50_ curves. Values above 50% cell death were chosen for each drug, where 50 nM and 100 nM BPD were above 50% viability, and 2.5 uM and 5 uM CED displayed >50% cell survival, according to the observed IC_50_ values. The individual drugs were tested in U87 cells in free and NP, and compared to see how nanoencapsulation affects cell viability. Statistical significance, as shown in Figure 8A, was found for cell death comparison between 50 nM and 100 nM NP BPD in the light toxicity condition (** *p* < 0.01). The improvement in BPD efficacy was further demonstrated by comparing cell viability decreases, 27% and 52% for dark versus light toxicity for 50 nM and 100 nM NP BPD, respectively. It should be noted that the reduction in cell viability to approximately 44% with 100 nM NP BPD resulted in this concentration not being used to assess co-administration effects, Figure 8C. There was approximately 21% increased cell death caused by 100 nM NP BPD, compared to 50 nM BPD with light toxicity, shown by cell viability averages of 65.4% and 44.4%, respectively. The effects of CED tested at 2.5 uM and 5 uM are shown in Figure 8B, where statistical significance for cell death was found in dark toxicity testing of 5 uM free drug (* *p* < 0.05), compared to that of light toxicity using 5 uM free CED. CED did not cause significant cell death in the NP form at the concentrations tested. The co-administration of BPD and CED was designed to test simultaneous administration of either both free or NP formulations at concentrations which resulted in above 50% cell viability, with a fixed dose of 50 nM BPD. The results shown in Figure 8C demonstrated statistically significant cell death when comparing 2.5 uM CED + 50 nM BPD in both free or NP whereby * *p* < 0.05. The NPs co-administered design yielded approximately 5% increased cell death over the free form co-administration of 2.5 uM CED + 50 nM BPD.

**Coefficient of Drug Interaction:** The results of the cell viability outcomes were followed by investigating the coefficient of drug interaction (CDI), shown in Table 4 below. The CDI values showed antagonistic effects in dark toxicity testing of co-administered 5 uM free CED + 50 nM free BPD, while synergistic effects were displayed in light toxicity testing of 2.5 uM CED + 50 nM BPD, both free and NP form, as well as less synergistic activity with 5 uM free CED + 50 nM free BPD. The CDI values were otherwise additive for all other treatment groups tested for co-administered, including dark toxicity testing of free and NP 2.5 uM CED + 50 nM BPD. Additive effects were also found in 5 uM NP CED + 50 nM NP BPD for both dark and light toxicity conditions.

## 4. Discussion

In order to address the unmet need in the treatment of GBM, we designed a study to test a combination therapy using BPD and CED in U87-MG cells. Other studies have encapsulated BPD and shown improved cellular uptake and subsequent cell death to support our findings [27,44]. A key unique portion of the presented study is the encapsulation of CED which has never been reported, and, of particular interest in terms of biocompatibility, using polymeric encapsulation. CED has been used widely for applications in GBM, ovarian cancer and sarcoma, for example, both in combination with other drugs, as well as monotherapy [39,57,58,59,60]. A phase III clinical trial for recurrent GBM failed due to no significant difference in progression-free survival for monotherapy or combination treatments [43]. Clinical trial outcomes could be improved by using an encapsulated form of the drug to extend the circulation time in the body and reduce undesirable side effects [42,43]. In our study, BPD and CED were encapsulated in mPEG-PLGA nanoparticles. Surface modification of nanoparticles with PEG also improves the colloidal stability of nanoparticles allowing them to be stored in solution prior to use [61]. The use of combination therapy to improve survival outcomes as well as previous in vitro studies investigating synergistic drug combinations in GBM inspired us to design a drug delivery system which addresses common problems such as MDR and poor bioavailability [29,33,41,62]. The aims of this research were to: (1) successfully synthesize reproducible batches of CED as well as BPD encapsulating mPEG-PLGA NPs with desirable size, PDI, zeta potential and encapsulation efficiency values, and (2) perform in vitro testing using non-encapsulated and NP encapsulated BPD and CED. The sizes of the synthesized NPs were within the acceptable range, under 200 nm, which have been shown to cross the BBB [63,64]. Moreover, the PDI values indicated monodispersity, and the largely negative zeta potentials inferred stable, non-aggregating NPs [65]. Electron microscopy images showed that the NPs had spherical morphology. Moreover, the images verified the size obtained from the DLS measurements, while also confirming the monodispersity of the NP population. After achieving reproducible synthesis, the NPs were tested to see if they were stable under physiological conditions. There were expected size variations found using TEM/SEM because of the preparation process whereby nanoparticle suspension samples are left to dry on carbon grids or silica wafers, respectively. The sizes found through DLS are respective to the distribution of hydrodynamic nanoparticle diameter, unlike TEM/SEM images that yield the particle diameter. The sizes of drug-loaded nanoparticles compared to empty nanoparticles are typically larger, as also found in previous studies done in our laboratory using similar nanoparticle preparation using BPD nanoparticles [1]. The high encapsulation of both drugs may have caused the slight increase in size compared to the empty nanoparticles [2]. In addition, the hydrophobic nature of the encapsulated drugs, CED and BPD, may cause swelling of the nanoparticles by interacting with the hydrophobic methyl groups of lactide in PLGA chains, electrostatic interactions and van der Waal’s forces within the amorphous nanoparticle core [2,3].

Significant findings from stability studies related to the physicochemical properties of polymeric NPs including zeta potential and release are related to the characteristics of encapsulated BPD and CED. CED NPs may have increased in size quickly over time due to the interactions of the drug with the polymer, aqueous environment with the nanoformulation and localization of CED within the nanoparticles. CED may adsorb on the external and/or towards the inner periphery of the nanoparticle, increasing its interaction with the components of media causing an increase in size overtime [3]. The zeta potential variations tend to occur due to proteins in the environment, where a protein corona forms around the NPs and causes the zeta potential to become less negative at initial time points. However, at later time points, these proteins dissociate and render the surface charge more negative once again [66,67]. Next, the drug release rate was studied using dialysis. The inherent characteristics of the drugs, such as hydrophobicity, concentration within the polymer matrix and molecular weight, all effect the rate at which the drugs interact and diffuse into the aqueous environment. The primary mechanism for drug release from a polymeric NP is via passive diffusion. BPD release from the NPs showed a biphasic profile with a prominent initial burst release followed by a gradual release reaching completion. BPD on the surface of the NP, will release quickly, diffusing from areas of high to low concentration, while content which is buried inside the amorphous polymeric chain networks of the NP takes longer to diffuse out of the NP. The drug release profiles may indicate that each drug has vastly different precipitation and degradation behavior. The release exponent show that BPD has Fickian diffusive properties, while CED has less-Fickian behavior. Following the release kinetic study, imaging and cell viability assays were conducted. Cellular uptake and observed differences in fluorescent intensity of BPD are important aspects of encapsulation benefits, as explained further.

The imaging results of this research showed how NP drug delivery enhances the resultant fluorescence in cells. Previous studies have shown that optimal BPD delivery from the NPs is observed within 30–60 min [44,68,69,70]. The variety of drug interactions found in this study provided a basis for understanding how CED and BPD cell viability effects are concentration-dependent, whereby additive, synergistic and antagonistic behaviors were observed according to viability and CDI results. This was found in our study, whereby a lower concentration of CED combined with BPD PDT revealed synergistic drug interactions in both free and NP, however NP yielded greater cell death. Other in vitro investigations have shown synergism when nanoparticle drug delivery was combined with PDT in U87 cells as well [71,72]. Antagonistic behavior at higher concentrations of CED may be attributed to CED quenching ROS produced by PDT. CED has an amine group in its structure which can act as electron donor and quench ROS, as other amine studies have shown [73,74]. Further studies to determine the effects of drug delivery order, including pre- and post-incubation with CED could enhance our understanding of drug interactions and optimal administration. Additionally, 3D cell models could also improve our understanding of how free versus NP CED and BPD co-administration therapy might work in vivo.

The study presented is applicable to the field of GBM treatment in that we have designed and implemented a unique combination of a remotely triggered light-sensitive agent which appeared to have enhanced cell killing with concomitant TKI administration. Superior tumor penetration and subsequent PS cytotoxicity of cancer cells may be possible with the use of polymeric encapsulation compared to free drug administration. Furthermore, concurrent use of encapsulated TKI agent which synergistically causes higher cytotoxicity of targeted tissue could reduce the required drug volumes used to treat GBM in patients.

## 5. Conclusions

Combination therapy for GBM offers a critical opportunity to improve outcomes in patient survival and quality of life with this detrimental disease. Remotely triggering agents that cause cytotoxicity in and around the tumor may help reduce the morbidities typically associated with chemotherapies [75]. All cytotoxic therapies result in pro-survival treatment responses like upregulated cytokine signaling from the tumor cells that often result in failure of the primary treatment and recurrence of disease [76]. Combining remotely triggered cytotoxic agents with drugs that can tackle these pro-survival treatment responses is helping reduce therapeutic outcomes in several in vitro and in vivo models of cancer [44,75,77,78]. Our understanding of the intricacies involved in effective removal and subsequent therapeutic administration of anti-cancer drugs to GBM tumors is that nanoencapsulated drug therapy may significantly enhance BBB penetration and cancer cell targeting using reduced drug concentrations required for efficacy, as well as side effects and inadvertent damage to healthy tissue. The use of a photosensitizing agent in combination with an antiangiogenic therapy is advantageous because of the ability to remotely trigger the PS while simultaneously halting cell proliferation via a secondary agent such as a pan-VEGFR tyrosine kinase inhibitor. By encapsulating each drug in this study, BPD, the PS, and CED, the cytostatic therapy, and investigating the effects of co-administration we found that synergistic effects occur, as opposed to the monotherapy cytotoxicity. Our investigation successfully synthesized, characterized, imaged and assessed the cell viability potential of polymeric pegylated BPD and CED NPs. Further testing in more intricate in vitro and in vivo models of GBM will help optimize this novel combination therapy. The results support using a co-administration therapy of BPD and CED in U87 monolayer as a basis for improving treatment methods for GBM.

## Figures and Tables

**Table 1:** Physicochemical Characterization of NPs (*n* = 4).

Characterization Parameter	Empty NPs	BPD NPs	CED NPs
Size (nm)	73.0 ± 7.2	88.5 ± 6.7	102.3 ± 7.6
PDI	0.13 ± 0.03	0.11 ± 0.01	0.09 ± 0.03
Zeta Potential (mV)	−29.1 ± 6.4	−24.1 ± 6.1	−16.3 ± 5.5
Encapsulation Efficiency (%)	No Drug	87.7 ± 7.4	78.2 ± 12.7

**Table 2:** Constants for drug release kinetics using Korsmeyer–Peppas model (*n* = 3).

	k_KP_	n	Time to 50% Drug Release (Hours)	Time to 80% Drug Release (Hours)
BPD	58.355	0.089	0.25	36
CED	31.592	0.462	2.6	7.5

**Table 3:** IC_50_ Values and Non-Linear Regression Analysis Results (*n* = 3).

	IC_50_	HillSlope Value	Equation of the Line	R^2^
BPD	172 nM	−1.2	Y = −9.875 + (101.6 + 9.875)/(1 + 10^((2.236 − x) * −1.2)	0.98
CED	6 uM	−2	Y = −5 + (91 + 5)/(1 + 10^((1 − x) * −2)	0.96

**Table 4:** Determining the Coefficient of Drug Interaction with Co-Administration of BPD and CED.

Co-Administration Combination	CDI (DT)	CDI (PDT)
Cf2.5 + Bf50	1.028	0.689
Cn2.5 + Bn50	1.048	0.793
Cf5 + Bf50	1.226	0.855
Cn5 + Bn50	1.029	1.040

CDI, coefficient of drug interaction (CDI < 1 synergistic, CDI = 1 additive, CDI > 1 antagonistic); DT, dark toxicity; PDT, photodynamic therapy; C, cediranib; B, bpd; f, free drug; n, NP formulation of drug; 2.5, 2.5 uM cediranib; 50, 50 nM bpd. CDI = AB/(A × B), where AB represents the ratio between the cell viability absorbance values of the co-administered drugs (CED + BPD) and control group, no treatment (dark toxicity), while A or B is the ratio between the cell viability absorbance values of each individual drug and the control group, no treatment (dark toxicity) [54].

## Supplementary Materials

The following are available online at http://www.mdpi.com/1999-4923/10/4/226/s1, Figure S1: Absorbance spectra data using ultraviolet-visible (UV-Vis) spectroscopy for CED (@328 nm) and BPD (@424 nm and 686 nm) in acetone. Dilution factor (DF) corresponds to the drug dilution in acetone, 401 (4 mL acetone + 10 uL BPD stock) and 2 (500 uL acetone + 500 uL CED stock), for BPD and CED, respectively, Figure S2: Chemical structures of CED (left) and BPD (right) as found in Pubchem data [79], Figure S3: Free vs Nano BPD imaging at 20x magnification at 60% light intensity (Cy5 filter) using various concentrations of BPD, including 50 and 250 nM not included in the main text of the document. Scale Bar: 200 um, Figure S4: The laser apparatus set up is shown (top) without laser on and (bottom) with laser on. The laser sensor (bottom picture with arrow) is used to confirm the power density of laser light (for example, 102 mW/cm2 as shown and used in this study for light irradiation). The 35-mm circular dish is placed on top of the dish holder (bottom picture with arrow) and light is directed from below the dish, and Figure S5: Stability study results at 24 hours post addition of media for CED NPs shown by SEM. CED NPs became unstable over the course of time points from 1 h to 24 h, shown in the main document in Figure 3. The SEM image at 24 hours supported the sizes found using DLS, found in Figure 3 of the main document, whereby the average NP size approached 200 nm. The NPs shown above are polydisperse as well.
