# Supplementary material for: Co-Administered Polymeric Nano-Antidotes for Improved Photo-Triggered Response in Glioblastoma
Source: Pharmaceutics. 2018 Nov 10;10(4):226. doi: 10.3390/pharmaceutics10040226 (PMC6321570; doi:10.3390/pharmaceutics10040226)
Supplement: Supplementary file 1 [file pharmaceutics-10-00226-s001.docx]

**Supplementary Materials: Co-Administered Polymeric Nano-Antidotes for Improved Photo-Triggered Response in Glioblastoma**

Janel Kydd, Rahul Jadia and Prakash Rai


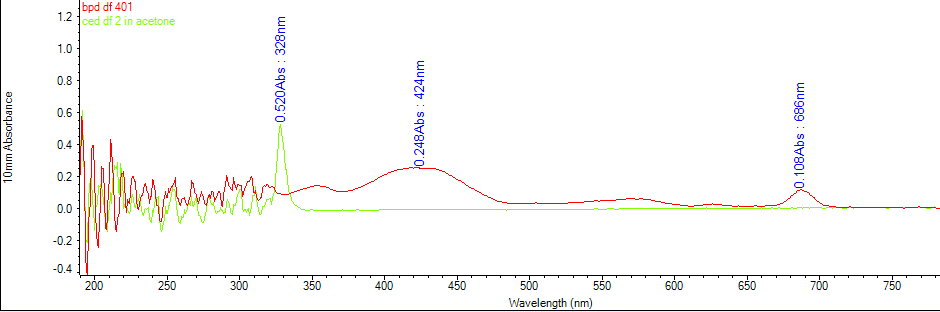


**Figure S1.** Absorbance spectra data using ultraviolet-visible (UV-Vis) spectroscopy for CED (@328 nm) and BPD (@424 nm and 686 nm) in acetone. Dilution factor (DF) corresponds to the drug dilution in acetone, 401 (4 mL acetone + 10 uL BPD stock) and 2 (500 uL acetone + 500 uL CED stock), for BPD and CED, respectively.


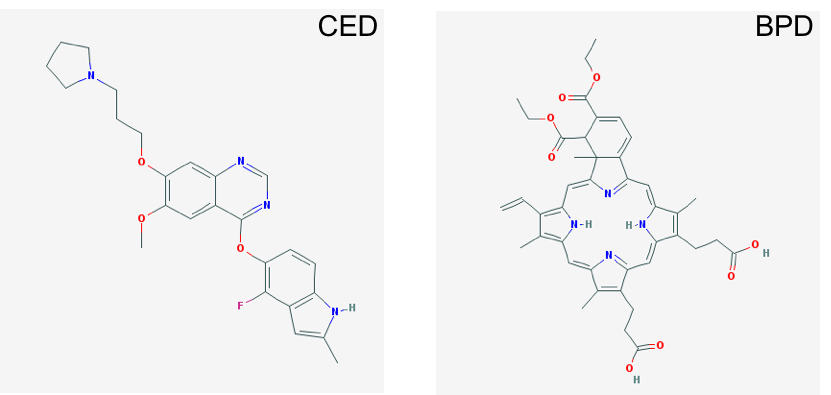


**Figure S2**. Chemical structures of CED (left) and BPD (right) as found in Pubchem data [79].


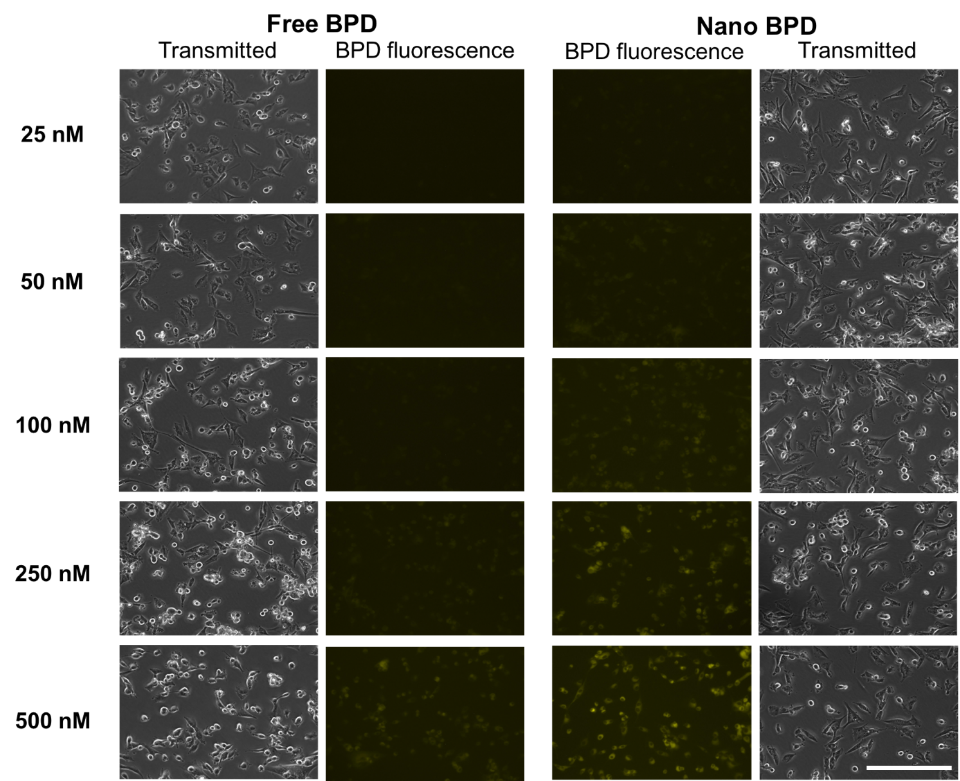


**Figure S3.** Free vs Nano BPD imaging at 20x magnification at 60% light intensity (Cy5 filter) using various concentrations of BPD, including 50 and 250 nM not included in the main text of the document. Scale Bar: 200 um.

**Figure S4.** The laser apparatus set up is shown (top) without laser on and (bottom) with laser on. The laser sensor (bottom picture with arrow) is used to confirm the power density of laser light (for example, 102 mW/cm^2^ as shown and used in this study for light irradiation). The 35-mm circular dish is placed on top of the dish holder (bottom picture with arrow) and light is directed from below the dish.


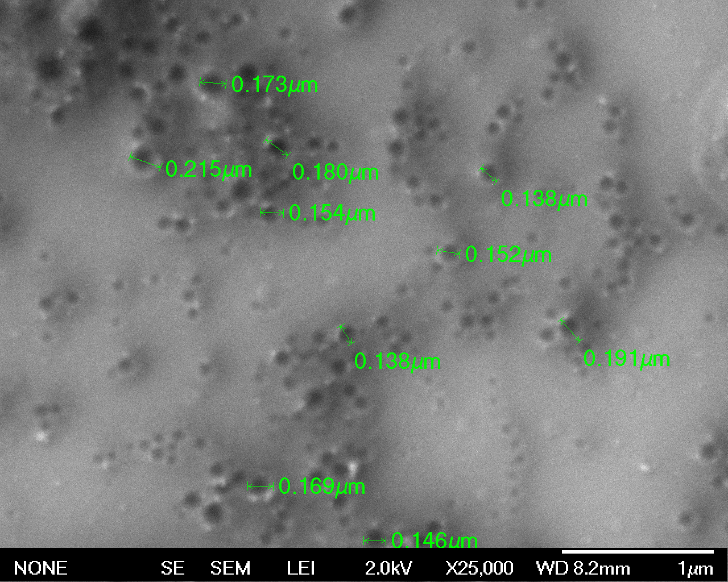


**Figure S5.** Stability study results at 24 hours post addition of media for CED NPs shown by SEM. CED NPs became unstable over the course of time points from 1 hour to 24 hours, shown in the main document in Figure 3. The SEM image at 24 hours supported the sizes found using DLS, found in Figure 3 of the main document, whereby the average NP size approached 200 nm. The NPs shown above are polydisperse as well.
